# Supplementary material for: Antioxidant and Photoprotective Activities of Viola philippica Polyol Extracts
Source: Antioxidants (Basel). 2025 Jul 18;14(7):884. doi: 10.3390/antiox14070884 (PMC12292903; doi:10.3390/antiox14070884)
Supplement: Supplementary file 1 [file antioxidants-14-00884-s001.zip › antioxidants-3716211-supplementary.pdf]

Antioxidant and Photoprotective Activities of *Viola philippica* Polyol Extracts

Jiang Li [1,2,†], Jiancheng Ma [1,3,†], Ya Li [1], Lan Luo [1], Wenhuan Zhang [4,5], Yong Tian [4,5], Yuncai Tian [4,5], Yi Li [1], Zhongjuan Wang [1,2,3] and Mingyi Wu [1,2,*]

[1] State Key Laboratory of Phytochemistry and Natural Medicines, Kunming Institute of Botany, Chinese Academy of Sciences, Kunming 650201, China; lijiang@mail.kib.ac.cn (J.L.); liya@mail.kib.ac.cn (Y.L.); luolan@mail.kib.ac.cn (L.L.); liyi@mail.kib.ac.cn (Y.L.)
[2] University of Chinese Academy of Sciences, Beijing 100049, China
[3] School of Pharmacy, Dali University, Dali 671003, China; majiancheng@mail.kib.ac.cn (J.M.); wangzhongjuan22@mails.ucas.ac.cn (Z.W.)
[4] Shanghai Zhenchen Cosmetics Co., Ltd., Shanghai 201415, China; zhangwenhuan@a-hcosmetics.com (W.Z.); tianyong@a-hcosmetics.com (Y.T.); tianyuncai@a-hcosmetics.com (Y.T.)
[5] Shanghai Zhizhenzhichen Technology Co., Ltd., Shanghai 201109, China
* Correspondence: wumingyi@mail.kib.ac.cn
† These authors contributed equally to this work.

Chromatographic conditions:

Column: Agilent ZORBAX SB-C18 (250 mm × 4.6 mm, 5 μm)

Gradient:

Time (min)	acetonitrile	0.1% phosphoric acid solution
0-25	8	92
25-40	8-60	92-40

Injection volume: 10 μL

Flow rate: 0.1 mL/min

Column temperature: 30°C

Detector wavelength: 344 nm

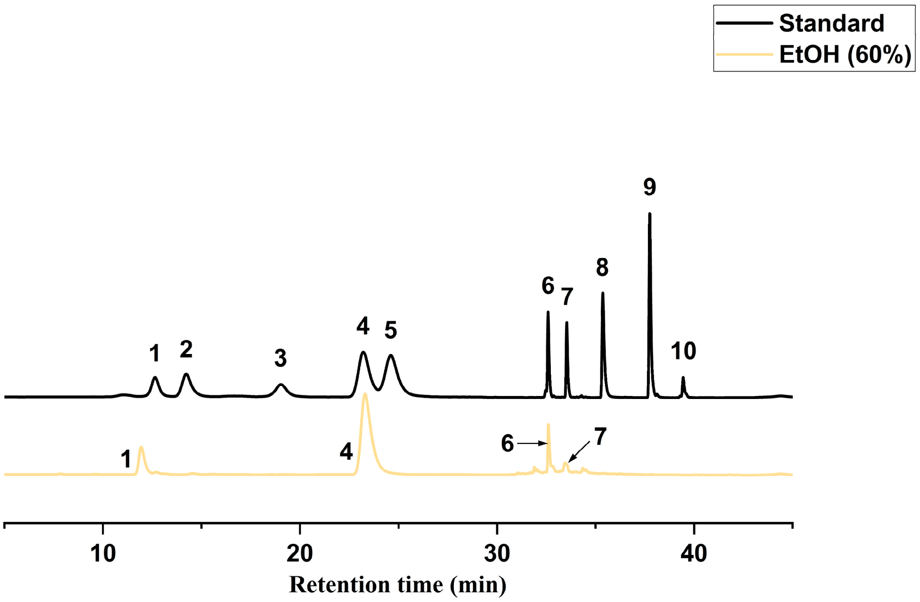

Figure S1. HPLC of VP ethanol extracts. 1. esculin, 2. caftaric acid, 3. chlorogenic acid, 4. esculetin, 5. caffeic acid, 6. schaftoside, 7. rutin, 8. chicoric acid, 9. luteolin, 10. naringenin.

Mass spectrometry conditions:

ESI Mode: The electrospray ionization (ESI) source was operated in both positive and negative modes.

Scan Range: 20~2000 m/z

Collision Energy: 10 V

Fragmentor Voltage: 135 V

Mode	Compound	Mass	Score	m/z	RT (min)
+ESI	Esculin	340.079	96.87	341	8.0076
	Esculetin	178.0264	99.73	179	16.0129
	Schaftoside	564.1478	99.30	565	29.5373
-ESI	Esculin	340.0795	99.86	339	8.0940
	Esculetin	178.0271	97.82	177	16.0330
	Schaftoside	564.1493	94.15	563	29.6127

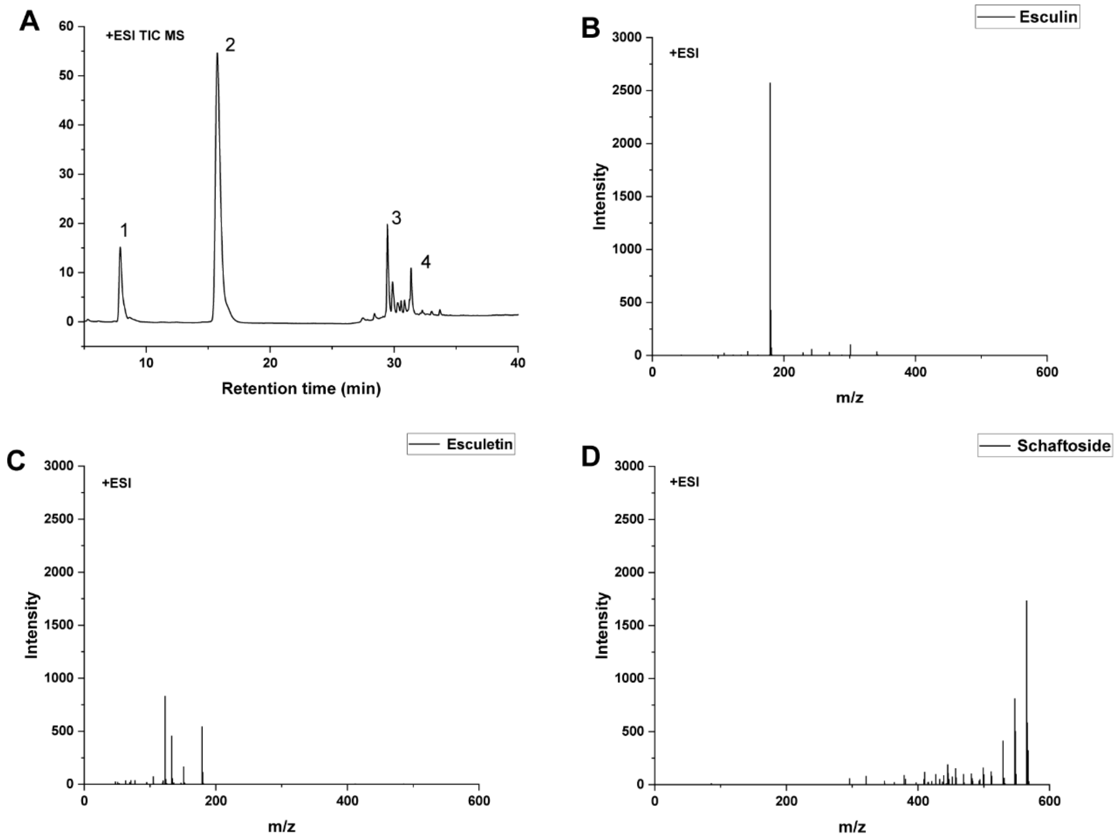

Figure S2. The LC/MS result of VP extract. (A) Mass chromatogram of the extract at positiveion mode; (B) mass spectrum of esculin (+ESI); (C) mass spectrum of esculetin (+ESI); (D) mass spectrum of shaftaside (+ESI). 1. Esculin. 2. Esculetin. 3 shaftaside. 4. Rutin.

Table S1. Calibration curves of esculin, esculetin, shaftaside, and rutin

Compounds	Calibration equation	R^2	LOD (μg/mL)	LOQ (μg/mL)
esculin	y = 15.136x + 15.105	0.9997	0.441	0.818
esculetin	y = 37.184x - 5.575	0.9999	0.642	2.740
shaftaside	y = 18.365x + 19.154	0.9999	0.127	0.779
rutin	y = 10.96x + 13.318	0.9999	0.275	0.620

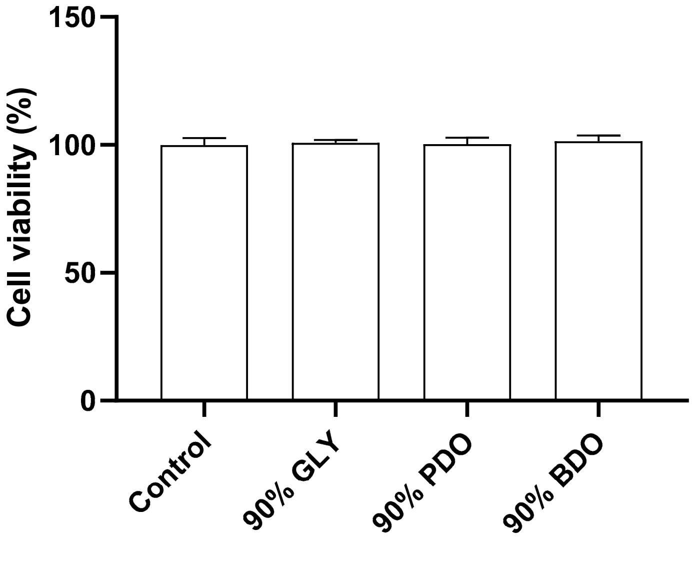

Figure S3. The effect of polyol solvents on HaCaT cell viability. Date were presented as mean ± SD (n = 3)

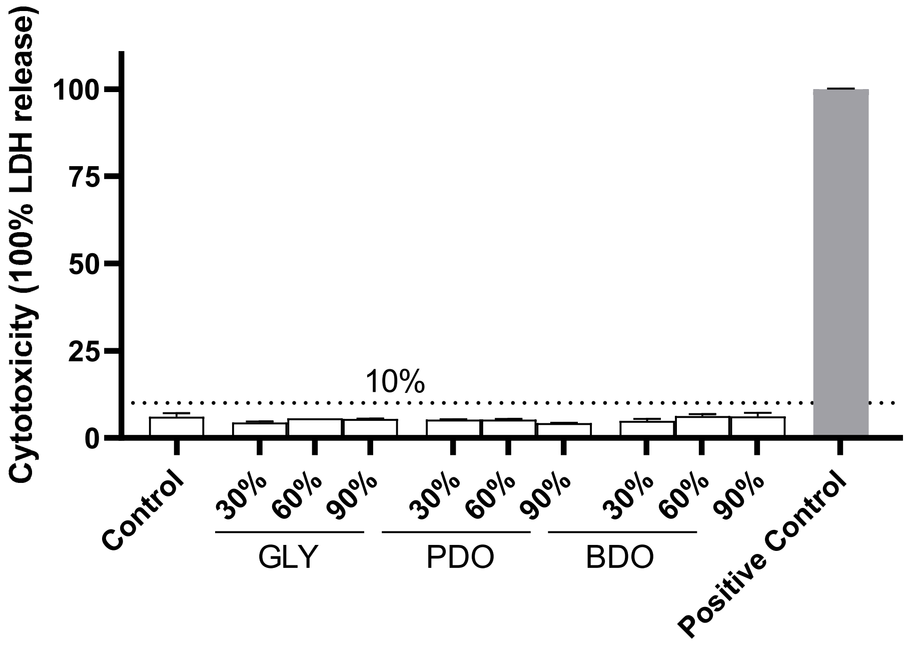

Figure S4. LDH release assay to determine cell death in HaCaT cells treated with VP extracts. Date were presented as mean ± SD (n = 3)

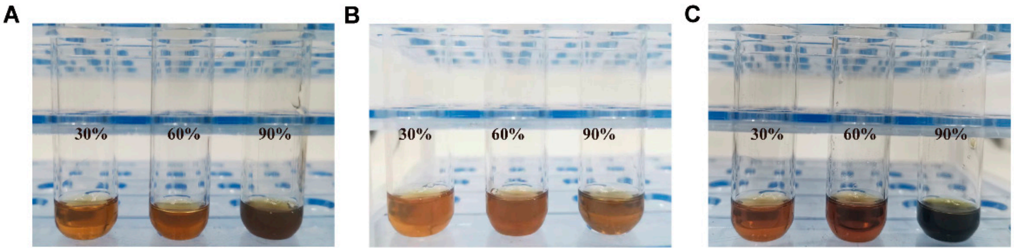

Figure S5. Colour and solubility of VP extracts. (A) Glycerol; (B) 1,3-propanediol; (C) 1,3-butanediol

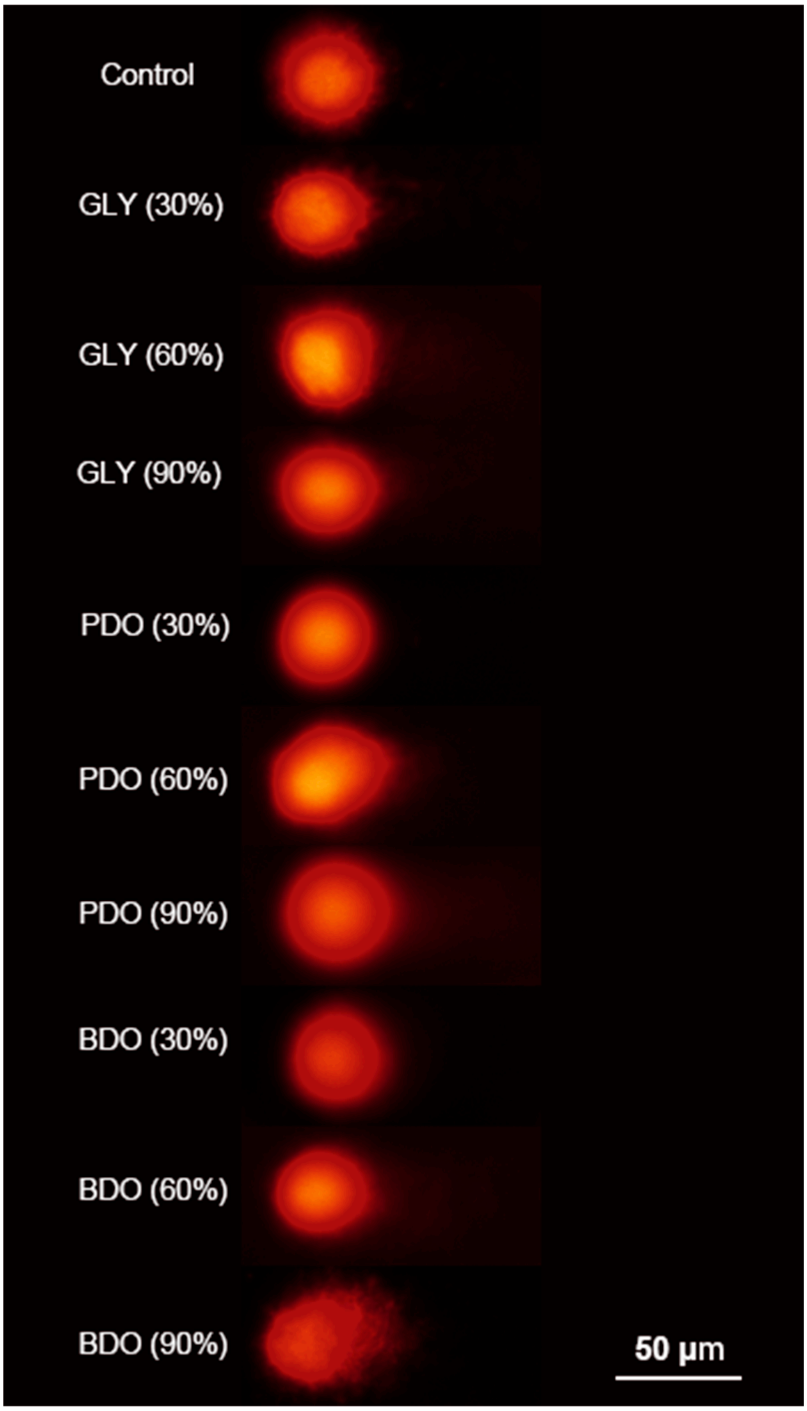

Figure S6. Effect of VP extracts on DNA damage in HaCaT cells
